# Passive Smoking as a Risk Factor of Dry Eye in Children

**DOI:** 10.1155/2012/130159

**Published:** 2012-07-31

**Authors:** Amany Abdel-Fattah El-Shazly, Walid Mohamed Abd El Raouf El-Zawahry, Ahmad Mohamed Hamdy, Manal Basyouni Ahmed

**Affiliations:** ^1^Department of Ophthalmology, Faculty of Medicine, Ain Shams University, Cairo, Egypt; ^2^Department of Pediatrics, Faculty of Medicine, Ain Shams University, Cairo, Egypt; ^3^Department of Biochemistry, Faculty of Medicine, Ain Shams University, Cairo, Egypt

## Abstract

*Purpose*. Adult active smoking is a risk factor for dry eye. We hypothesize that passive smoking in children can also produce the same effects. *Methods*. We included 112 school children presenting with eye discomfort. Assessment of eye dryness and its severity levels depending on symptoms of dry eye, visual symptoms, tear breakup time (TBUT), Schirmer-1 test, and corneal fluorescein staining were done for all of them. Exposure to cigarette smoking was assessed by history-taking and urinary cotinine levels. *Results*. Dry eye was found in 80/112 children. Passive smoking was documented in 76/112 children. Number of cigarettes to which the child was exposed per day and the duration of exposure to passive smoking were significantly higher in children with dry eye compared to those without. Urinary cotinine, and cotinine/creatinine ratio (CCR) was significantly higher in children with dry eye compared to those without dry eye. Multiregression analysis showed that the most important determinants of dry eye were CCR and number of cigarettes/day. *Conclusion*. Passive smoking represents a significant risk factor of dry eye in children comparable to that shown with active adult smoking. Male children are more prone to this effect.

## 1. Introduction

Dry eye is a multifactorial disease of the tears and ocular surface that results in symptoms of discomfort, visual disturbance, [1–3], and tears film instability with potential damage to the ocular surface. It is accompanied by increased osmolarity of the tear film and inflammation of the ocular surface [4, 5]. 

Chronic exposure to cigarette smoke is harmful to ocular tissues through ischemic or oxidative mechanisms [6]. Smoking cigarettes also increases the risk of dry eye syndrome and exacerbates existing conditions [7].

This study aimed to investigate the relation between passive smoking and dry eye in children.

## 2. Subjects and Methods

This cross-sectional clinical study was carried out from June 2008 to December 2009. We started with 300 children (5–12 years old) presenting with eye discomfort to the ophthalmology clinics. The hospital serves patients from eastern Cairo which has the same degree of social and environmental status. After exclusion of a big list of direct causes of eye discomfort (188 patients), the remaining 112 children (60 males and 52 females with a mean age of 7.28 ± 1.31 for males and 7.51 ± 1.25 years for females) were assessed for eye dryness both clinically and by tests for tear film status. This group of children was then classified into two major groups: group 1 without eye dryness (32 patients) and group 2 with eye dryness (80 patients). Passive smoking was assessed in the 112 children based on history of exposure and confirmed with urinary cotinine and urinary cotinine creatinine values.

Exclusion criteria are

1. active smokers;
2. conjunctivitis;
3. contact lens users;
4. history of ocular surgery in the last 6 months;
5. systemic diseases such as diabetes mellitus and collagen disorders;
6. those with atopy or allergic diseases;
7. drugs as antihistaminics and atropine with its similar agents.

Group 1 without eye dryness (32 patients) comprised 14 patients with hypermetropia, 8 patients with astigmatism, 6 patients with emotional instability, and 4 patients with sleep disturbances. Correction of the underlying cause was followed by disappearance of the eye discomfort in all cases with errors of refraction, 3 cases with emotional instability that was associated with other depressive manifestations who were referred to pediatric psychiatrist (mother death in one patient, separation of parents in 2 patients), and 2 cases with sleep disturbances (inverted sleep rhythm and short interval of sleep).

After approval of the local ethics committee, informed consents were taken from parents or guardians of those children. 

All included children were subjected to detailed history taking with special emphasis on pattern and degree of exposure to smokers. Past history of recurrent conjunctivitis, respiratory allergy, or infections was taken as well. Detailed ocular history was taken including foreign body sensation (sandy), irritation, discomfort, eye itching, and discharge. Medical examination was done to assess general condition and respiratory manifestations. We looked at nyctalopia, hemeralopia, or xerophthalmia as signs of vitamin A deficiency [8].

Ophthalmologic evaluation included full assessment with focus on tests for ocular dryness. The tests were carried out in sequence, starting with tear film (TBUT) done for three times with calculation of mean of the three readings [9, 10]. The next test was examination of the cornea by fluorescein staining (Table 1) with scoring according to [11]. The last test was estimation of basic secretion using Schirmer-1 test without anesthesia. This sequence of testing was adopted to minimize any error due to reflex tearing [9, 10]. Breakup time test was done by putting a fluorescein filter paper in the lower fornix and asking the patient to blink, the interval between the last complete blink and the appearance of the first corneal black spot in the stained tear film was measured three times, and the mean value of the measurements was calculated. Schirmer-1 test was done without topical anaesthesia bilaterally, by the standardized strips of filter paper placed in the lateral canthus away from the cornea and left in place for 5 min with the eyes closed. Readings were recorded in millimetres of wet strip. A TBUT value of less than 10 seconds and Schirmer-1 test value of less than 5 mm were considered as abnormal. The results of these tests were scored according to (Table 1).

## 3. Statistical Analysis

We conducted statistical analysis using the statistical package for the social sciences (SPSS software, version 15). Quantitative variables were expressed as mean ± SD, and whereas qualitative variables were given as numbers and percentage. Student's *t*-test was used to assess the statistical significance of differences between two groups. Descriptive statistics was done using chi-square analysis. Correlation analysis was performed by calculating Pearson correlation coefficient (*r*). Regression analysis was done to assess the different factors that can increase the risk of dry eye.

## 4. Results

Dry eye was found in 80 children among 112 ones presenting with symptoms suggestive of dryness. The present study showed that age and body mass index were not significantly different between children with dry eye and those without dryness.

The passive smoking was documented in 62 male and 14 female children with an age range of 6–12 years (7.35 ± 1.25 years). The nonexposed group comprised 18 male and 18 female children with an age range of 5–12 years (7.31 ± 1.22 years). 

The number of smoked cigarettes to which the child was exposed per day and the duration of exposure of the child to passive smoking was significantly higher in children with dry eye (17.70 ± 14.19 and 10.00 ± 3.77 hours, resp.) compared to those without (0.652 ± 2.55 and 0.70 ± 2.38 hours, resp.) (*P* value of < 0.0001 for both). Urinary cotinine and cotinine/creatinine ratio was significantly higher in children with dry eye (66.64 ± 15.71 *μ*g/L and 210.17 ± 48.36 ng/mg, resp.) compared to those with nondry eye (15.03 ± 8.11 *μ*g/L and 50.62 ± 22.72 ng/mg, resp.) (*P* values of < 0.0001) (Table 2 ).

Dry eye was more commonly encountered among male children than females (0.003). Signs of vitamin deficiency were not significantly different between children with dry eye and those without (Table 3). Moreover, passive smoking in the males with eye dryness was more intense than in females with CCR being significantly higher in males (260.12 ± 56.14 ng/mg) than in females (192.56 ± 23.45 ng/mg) with a *P* value < 0.001.

Dry eye score showed significant positive correlation with the number of cigarettes, duration of smoking, urinary cotinine and urinary cotinine/creatinine ratio. However, it was not significantly correlated with age or body mass index (Table 4).

Multiregression analysis (*R* ^2^ of 0.827) showed that the most important determinants of dry eye in this study was CCR (beta = 0.786 and *P* = 0.006) and number of cigarettes per day (beta = 0.337 and *P* = 0.002) (Table 5). 

## 5. Discussion

The diagnosis of dry eye was confirmed in the majority of children presenting with symptoms of dry eye. Dry eye disease in children can occur in association with a number of congenital, autoimmune, endocrine, and inflammatory disorders, or under certain environmental and nutritional conditions [12].

Dry eye was more commonly encountered among male children than females (*P* = 0.003). 

Body mass index as a reflection of the nutritional status was not different between children with dry eye compared to those without. Similarly, signs of vitamin deficiency were not significantly different between children with dry eye and those without.

The urinary cotinine and cotinine/creatinine ratio made an objective validation of the history of passive smoking. The credibility of these parameters was ensured in a previous work [13]. In the present study, 76 patients were exposed to smoking, and their passive exposure to cigarette smoking was proved by the high values of urinary cotinine and urinary cotinine/creatinine ratio. 

Passive smoking was more common in male children than female ones. This male preponderance was noticeable as well with dry eye. This may be attributed to the common habit that males usually join adults more frequently than female children in our community.

Passive smoking assessed as number of smoked cigarettes near to the child per day and the duration of exposure of the child to passive smoking as well as laboratory findings of urinary cotinine and cotinine/creatinine ratio showed higher results in children with dry eye compared to children without dry eye. Moreover dry eye score had significant positive correlation with smoking parameters.

The multiregression analysis showed that passive smoking represented by CCR and number of cigarettes per day was the most important determinant of eye dryness in this group of children.

These results are supplemented by the work of Matsumoto et al. [14] who found that TBUT was 11.9±5.8 seconds in active smokers and 14.9±5.5 in nonsmokers. Moreover, they found that Schirmer-1 test results were lower in smokers compared to nonsmokers. Similarly, Altinors et al. [15] found that the mean TBUT was 5.3 seconds and the mean Schirmer-1 test value was 10.8 mm in active smokers, which were significantly lower than those values of nonsmokers. Yoon et al. [16] also found that tear film BUT and basal tear secretion in smokers were significantly lower than in nonsmokers. Matsumoto et al. [14] reported similar results.

Regarding the fluorescein staining score, Yoon et al. [16], contrary to our results, found that fluorescein staining score in smokers (0.40 ± 0.77) was not different from that of controls (0.38 ± 0.80).

Although there are no previous reports concerning relation of passive smoking to the eye dryness of children, yet many studies on adults showed that active smoking is a significant risk factor for eye dryness whether done on general population or performed as hospital-based ones [17–23].

We can conclude that passive smoking is a commonly encountered risk factor of dry eye among the young children. Males are more prone for this effect.

## Figures and Tables

**Table 1:** Dry eye severity grading scheme.

Dry eye severity level	1	2	3	4*
Discomfort, severity and frequency	Mild and/or episodic; occurs under environmental stress	Moderately episodic or chronic, stress or no stress	Severely frequent or constant without stress	Severe and/or disabling and constant
Visual symptoms	None or episodic mild fatigue	Annoying and/or activity-limiting episodic	Annoying, chronic and/orconstant, limiting activity	Constant and/or possiblydisabling
Conjunctival injection	None to mild	None to mild	±	+/++
Conjunctival staining	None to mild	Variable	Moderate to marked	Marked
Corneal staining (severity/location)	None to mild	Variable	Marked central	Severe punctate erosions
Corneal/tear signs	None to mild	Mild debris and ↓ meniscus	Filamentary keratitis, mucus clumping, and ↑ tear debris	Filamentary keratitis, mucus clumping, and ↑ tear debris, and ulceration
TFBUT (sec)	Variable	≤10	≤5	Immediate
Schirmer score (mm/5 min)	Variable	≤10	≤5	≤2

^∗^Must have signs and symptoms; TBUT: fluorescein tear breakup time; reprinted with permission from Behrens A, Doyle JJ, Stern L et al. Dysfunctional tear syndrome. A Delphi approach to treatment recommendations (Cornea 2006; 25: 90–7).

**Table 2:** Age, body mass index, and smoking parameters between children with dry eye compared to those without dry eye.

Parameter	Nondry eye group (32)	Dry eye group (80)	*t*	*P*
	Mean ± SD	Mean ± SD		
Age (years)	7.91± 2.63	6.9 ± 2.29	−1.35	0.19
Body mass index	23.30 ± 2.99	23.09 ± 3.74	−0.22	0.83
Number of cigarettes per day	0.652 ± 2.55	17.70 ± 14.19	5.40	<0.0001
Duration of smoking exposure (hours /day)	0.70 ± 2.38	10.00 ± 3.77	8.45	<0.0001
Urinary cotinine levels (*μ*g/L)	15.03 ± 8.11	66.64 ± 15.71	14.22	<0.0001
Urinary cotinine/creatinine ratio (ng/mg)	50.62 ± 22.72	210.17 ± 48.36	14.62	<0.0001

**Table 3:** Gender, vitamin deficiency, and indoor smoking between dry eye and nondry eye groups.

	Patient with nondry eye	Patient with dry eye	Chi test	*P*
Males	17	63	9.09	0.003
Females	16	16		
Smoking positive	6	70	49.53	<0.0001
Smoking negative	26	10		
Positive signs of vitamin deficiency	7	12	0.77	0.381
Negative signs of vitamin deficiency	25	68		

**Table 4:** Correlations between score of dry eye with smoking parameters in dry eye group.

	Dry eye score	
	*r*	*P*
Age	−0.21	*P* = 0.16
Body mass index	0.13	*P* = 0.39
Number of cigarettes per day	0.74	*P* < 0.0001
Duration of smoking exposure	0.68	*P* < 0.0001
Cotinine	0.84	*P* < 0.0001
Cotinine/creatinine ratio	0.88	*P* < 0.0001

**Table 5:** Multiple regression analysis of different factors.

	BETA	*t*	*P*-level
Age	−0.020	−0.285	0.777
BMI	0.059	0.811	0.422
Vitamine deficiency	−0.022	−0.314	0.755
Duration of smoking	−0.025	−0.219	0.827
Number of cigarettes per day	**0.337**	**3.251**	**0.002**
Cotinine	−0.119	−0.417	0.679
Cotinine/creatinine ratio	**0.786**	**2.916**	**0.005**

*R* = 0.909, *R* ^²^ = 0.827, Adjusted *R* ^²^ = 0.796, *F* = 26.638, *P* < 0.00000, sd. error of estimate: 0.469.

BMI: body mass index.
